# Reactivating Sleeping Intramedullary Nail in a 16-Year-Old Female with Polyostotic Fibrous Dysplasia: A Case Report on Complications and Potential Solutions

**DOI:** 10.3390/life14121543

**Published:** 2024-11-25

**Authors:** Marco Todisco, Marianna Viotto, Laura Campanacci, Giovanni Luigi Di Gennaro, Alessandro Depaoli, Gino Rocca, Giovanni Trisolino

**Affiliations:** 1 Pediatric Orthopedics and Traumatology, IRCCS Istituto Ortopedico Rizzoli, 40136 Bologna, Italy; marco.todisco@ior.it (M.T.); marianna.viotto@ior.it (M.V.); giovanniluigi.digennaro@ior.it (G.L.D.G.); gino.rocca@ior.it (G.R.); 2 Department of Orthopedic Oncology, IRCCS Istituto Ortopedico Rizzoli, 40136 Bologna, Italy; laura.campanacci@ior.it; 3 Pediatric Orthopedics and Traumatology, IRCCS Istituto Ortopedico Rizzoli, 90011 Bagheria, Italy; alessandro.depaoli@ior.it

**Keywords:** fibrous dysplasia, polyostotic, lower limb length discrepancy, expandable intramedullary magnetic nail, reactivation, lengthening procedure

## Abstract

Background: Fibrous dysplasia (FD) is a rare condition in which normal spongy and cortical bone is replaced by non-neoplastic fibrous tissue, leading to weakened bone matrix and increased risk of pathological fractures and deformities. Treating these deformities poses a significant challenge for surgeons. While various cases of surgical stabilization and limb lengthening using intramedullary nails have been reported, there is limited evidence on the use of Motorized Intramedullary Limb-Lengthening Nails (MILLNs) in FD patients. This case report presents the clinical history of a patient with FD who underwent multiple surgical interventions to address severe lower limb length discrepancy (LLD) and angular deformity caused by multiple fractures. Case presentation: A sixteen-year-old Caucasian girl with polyostotic FD developed a severe post-traumatic LLD of 10 cm on the right side, associated with coxa vara, valgus knee, and patellar instability. The deformity of the proximal femur was addressed with a valgus and derotational femoral osteotomy. However, this procedure exacerbated the knee’s valgus deformity and only partially corrected the LLD, leading to the decision to proceed with femoral lengthening. A retrograde magnetic intramedullary nail (PRECICE, NuVasive) was utilized for this purpose. Approximately three months postoperatively, radiographs revealed the loosening of the proximal anchoring screw, while the nail had reached maximum distraction. We then proposed reactivating the previously implanted nail. Nine months after the final surgery, standing long-leg radiographs showed a residual shortening of 1 cm, with excellent healing at the fracture sites and the nail and screws remaining securely in place. The patient was monitored regularly, with the latest follow-up occurring four years and five months after the conclusion of the last lengthening procedure. Conclusions: This case report describes the reactivation of a MILLN in a patient with polyostotic fibrous dysplasia. While nail reactivation has been previously described in the literature, to our knowledge, it has not been reported for treating complications arising from FD. In cases of mechanical complications, this approach can equalize leg length discrepancies and correct deformities, avoiding additional invasive surgeries and reducing healthcare costs. As this is an off-label treatment, preoperative consent from both the patient and the parents is required.

## 1. Introduction

Fibrous dysplasia (FD) is a rare condition characterized by the replacement of normal spongy and cortical bone with non-neoplastic fibrous tissue. It results from a mutation in the GNAS gene (located on the 20q13.32 region of chromosome 20), which regulates bone formation through the cAMP signaling pathway [1]. This mutation occurs post-zygotically, resulting in a genetic mosaic. This means that within a single individual, or even within the same body segment, there are distinct populations of affected and unaffected cells. All of this is driven by phenotypic variability, which arises from a significant process of parental imprinting and post-zygotic mosaicism. Consequently, the mutation may only be detectable in certain cells, complicating genetic molecular diagnosis [2]. FD has no gender predilection, and its clinical presentation can occur at any age. FD can affect a single bone (monostotic) and multiple bones (polyostotic); it may also be associated with endocrinopathies and skin involvement in McCune–Albright Syndrome or with intramuscular mixomas in Mazabraud Syndrome [2,3]. In monostotic forms, the most frequently affected site is the proximal femur, leading to the characteristic ‘shepherd’s crook’ deformity. When the involvement is polyostotic, the tibia, humerus, ribs, clavicle, and facial bones are often affected. In cases associated with McCune–Albright Syndrome, the early development of secondary sexual characteristics and ‘café au lait’ spots on the patient’s trunk may also be observed [4]. Mazabraud Syndrome is an extremely rare condition, with just over 100 cases reported in the literature. It is characterized by the presence of typically polyostotic dysplastic lesions, usually presenting clinically around the age of 40, accompanied by the development of generally painless and benign intramuscular myxoma.

The clinical history of FD varies based on the nature of the disease. The monostotic form is often asymptomatic and may be discovered incidentally on radiographs performed for unrelated reasons. In contrast, the less common polyostotic form can lead to severe and progressive deformities, which may even develop after skeletal maturity, causing chronic pain, disability, and increasing the risk of pathological fractures [4]. In patients with McCune–Albright Syndrome, the initial symptoms may include early hormonal imbalances and accelerated growth, often accompanied by skin involvement presenting as café au lait macules [5]. Skeletal lesions in McCune–Albright Syndrome tend to be larger and can lead to major complications [4].

The fibro-osseous anomaly associated with FD interferes with bone maturation and development, leading to disorganized and inconsistent architectural characteristics, along with compromised integrity marked by notably low mineral density [1]. FD, by weakening the bone matrix, increases the risk of pathological fractures and deformities. The most significant among these involve the lower limbs and can lead to considerable LLD and malalignments that necessitate surgical intervention [2,4,6].

The treatment is determined by the location, the extent of the dysplasia, and the symptoms caused by the lesion. Monostotic forms and isolated asymptomatic lesions can be managed with simple observation and periodic monitoring. In cases of polyostotic forms, symptomatic lesions, or those affecting the lower limbs, surgical treatment is mandatory [7]. The treatment of fractures and deformities caused by FD presents a significant challenge for the surgeon. In FD, the bone is often weak and thin, compromising screw retention during fixation and increasing the risk of the early loosening of the hardware. For this reason, treatment with intramedullary nails is preferred over the use of plate and screw fixation [8].

Various cases of surgical stabilization and limb lengthening using intramedullary nails have been described in the literature [9,10,11,12], but there is limited evidence regarding the use of MILLN for FD [13]. Attempts to reuse the same extendable nail have been reported [14]. However, to our knowledge, there have been no documented cases involving the reactivation of MILLN for the treatment of discrepancies in patients affected by FD.

In this case report, we describe the clinical history of a patient affected by FD who underwent multiple surgical interventions to correct lower limb length discrepancy and angular deformity resulting from multiple fractures. Our aim is to emphasize the failures and complications encountered in treating this challenging condition and to detail the outcomes of possible solutions.

## 2. Case Presentation

**Figure 1:**
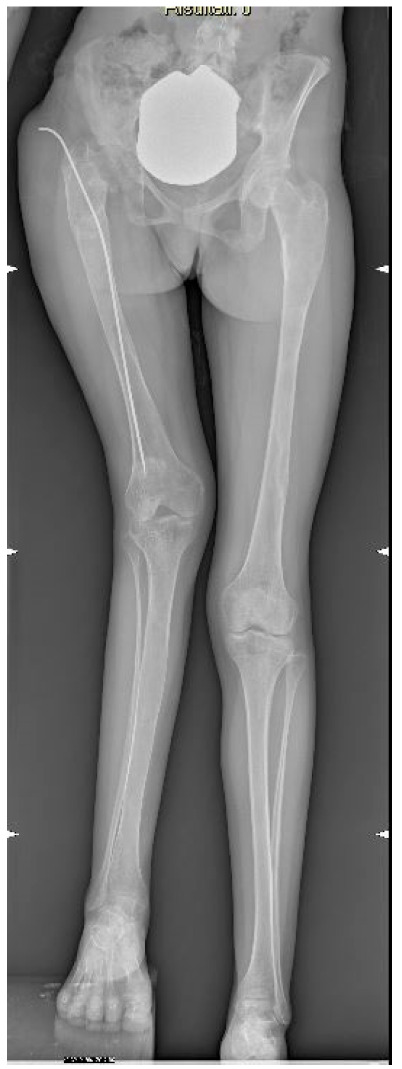
Patient X-ray: 10 cm of LLD, coxa vara and valgus knee.

A sixteen-year-old Caucasian girl with polyostotic FD developed a severe post-traumatic LLD of 10 cm on the right side, associated with coxa vara, valgus knee, and patellar instability (Figure 1).

**Figure 2:**
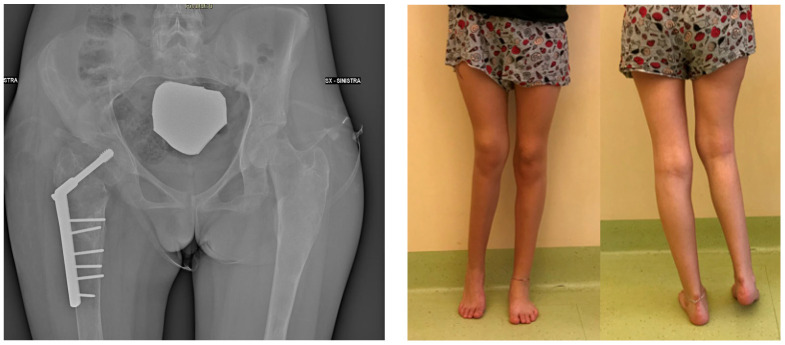
Postoperative X-ray and clinical pictures of the valgus and derotational femoral osteotomy.

The patient had no comorbidities and exhibited regular growth with no significant LLD or deformity during infancy. Between the ages of 10 and 15, she experienced three pathological fractures, including a proximal diaphyseal fracture of the right femur treated with an elastic intramedullary nail, a basicervical fracture, and an additional proximal diaphyseal fracture managed conservatively with a hip spica cast. These fractures resulted in a type 3 shepherd’s crook deformity according to Ippolito. During the surgical fixation of one of these fractures, a biopsy confirmed the diagnosis of FD, which had only been suspected up to that point. At 16 years and 3 months of age, the proximal femur deformity was treated with a valgus and derotational femoral osteotomy, combined with adductor tenotomy (Figure 2).

This procedure, while improving hip function, accentuated the valgus deformity of the knee and only partially reduced the LLD to approximately 8.5 cm, prompting the proposal for femoral lengthening. We decided to use a retrograde magnetic intramedullary nail (PRECICE, NuVasive, San Diego, CA, USA). An alternative method that could have lengthened the affected limb while also correcting the deformity was the use of a hexapodal external fixator. However, this option was dismissed to spare the patient, who had already undergone several surgeries, from enduring a lengthy postoperative recovery and living with an external fixator for months, especially since a less invasive option was available. The MILLN technique indeed presents a lower rate of complications (such as superficial infections), allows for a greater range of motion in the affected joint during both the lengthening and consolidation phases, and is also better tolerated by patients in daily life [15]. The case was thoroughly discussed with the parents, who were well informed about the risks and benefits of the treatment. Due to the presence of the plate at the proximal femur, it would not have been possible to introduce a sufficiently long nail to fully correct the LLD, as the distal femur could accommodate a nail that allows for a maximum lengthening of 5 cm. Despite this limitation, the patient and her parents agreed that the goal of the procedure was to aim for a 3 cm achievement in the LLD.

**Figure 3:**
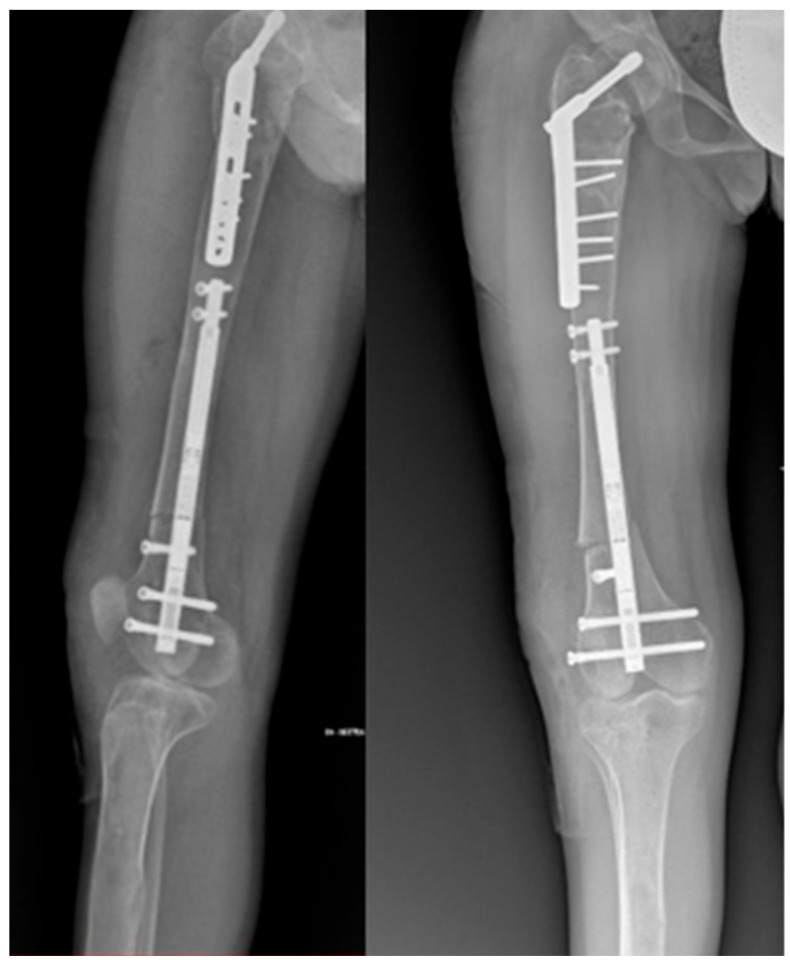
Postoperative X-ray with a retrograde magnetic intramedullary nail and a poller screw.

Thorough preoperative planning was conducted. By reviewing the X-rays, the diameter of the medullary canal and the distal portion of the proximal femur below the plate were measured to determine the appropriate size of the nail to be used. With the patient in a supine position under general anesthesia, a lateral parapatellar incision was made. Under fluoroscopic control, an osteotomy was performed at the level of the distal metaphysis, and a temporary external fixator was placed to correct the femoral axis. Subsequently, the retrograde magnetic intramedullary nail (diameter: 10.7 mm; length: 215 mm) was implanted and secured with two proximal and two distal screws. To adhere to the principles of the reverse planning method by Baumgart [16] and to address the valgus deformity of the distal femur (mLDFA = 84°, aLDFA = 77°, and MAD = 45 mm), a varus hypercorrection was stabilized with a lateral poller blocking screw (Figure 3). Finally, a release of the lateral retinaculum was performed.

The distraction started two days after surgery at a rate of 0.25 mm twice a day. We typically extend at a rate of 0.5 mm twice daily. This rate was selected for several reasons, including the compromised bone quality and the need to prevent additional luxations due to the instability of the patella. Weight bearing was not allowed during the lengthening phase, but exercises of active mobilization and of muscular strengthening were prescribed.

**Figure 4:**
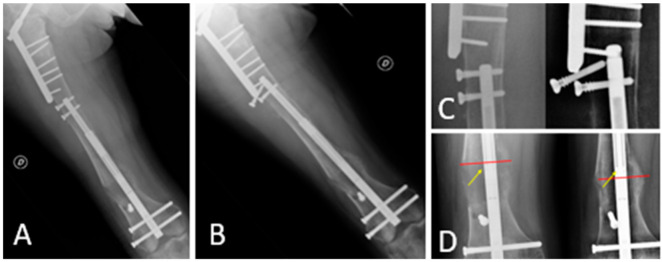
(**A**): Follow-up X-ray conducted 2.5-month postoperatively; (**B**): follow-up X-ray conducted 3-month postoperatively showing loosening of the proximal anchoring screw; (**C**): the upward migration of the nail, complete loss of the proximal screw, and partial loss of the other proximal screw, with the cortical bone that eroded; (**D**): the decreased height of the bone regeneration (Indicated by the red line), which is below the magnetic component (Indicated by the yellow arrow).

At 2.5 months postoperative, radiographs showed an elongation of approximately 35 mm with a callus exhibiting pattern 1 according to Li [17] and the distraction rate was lowered at 0.25 mm once a day. After three weeks, in the absence of specific symptoms, a scheduled follow-up radiographic examination was performed, which documented the subsidence of the nail with the proximal screws exhibiting a loss of fixation. The most proximal screw exhibited complete loosening, while the distal screw showed a rise of approximately 12 mm, corresponding to a reduction in the height of the regenerated bone at the lengthening site (Figure 4).

**Figure 5:**
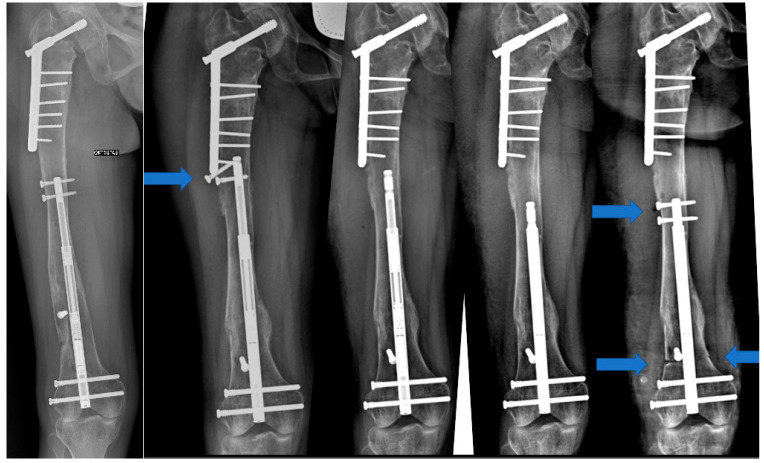
In sequence, we see the mobilization of the proximal screws (Indicated by the proximal arrow) and the subsequent surgical steps, the removal of the proximal screws, the retraction of the nail, the repositioning of the proximal screws, new osteotomy with reactivation of the nail, and the final regenerate (Indicated by the double distal arrows).

Eight months after nail implantation, the bone callus was almost completely formed, leading to the decision to allow partial weight bearing. After a detailed discussion with the parents and the patient, we proposed reactivating the previously implanted nail. A surgical procedure was planned to remove the proximal screws. After 5 days, once the complete retraction of the PRECICE nail was confirmed radiographically, the patient was taken back to the operating room. Through two longitudinal medial and lateral accesses to the distal femoral metadiaphysis, a new linear femoral osteotomy was performed using a Gigli saw, securing the nail proximally with two 30 mm screws (Figure 5).

The distraction rate was immediately resumed at 0.25 mm × 2 per day using dedicated machinery. At 2.5 months, a further 4 cm lengthening was achieved. A new distraction rate of 0.25 mm × 2 every other day was prescribed. At four months, the lengthening reached 5 cm, so it was stopped, and progressive weight bearing was allowed.

**Figure 6:**
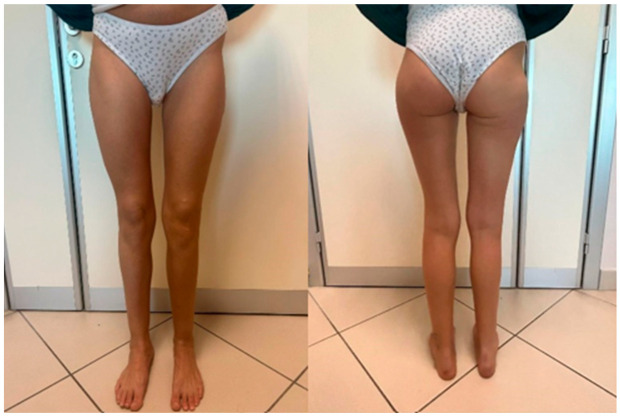
Clinical photo of the patient at a follow-up of 4.5 years. As can be seen from the gluteal folds, the length of the lower limbs was fully achieved despite the remaining 1 cm leg length discrepancy visible on the X-ray. However, there is still atrophy of the right gluteus and quadriceps compared to the contralateral side. The lower limbs are well aligned overall.

Nine months after the last surgery, a panoramic X-ray of the lower limbs showed a residual shortening of 1 cm, as well as the excellent healing of the fracture sites, with the nail and screws in place. The MAD (mechanical axis deviation) improved from 5.4 cm preoperatively to 1.9 cm postoperatively. The aLDFA (anatomical lateral distal femoral angle) increased from 77.9° preoperatively to 86.5° postoperatively. The patient was monitored until the latest check for a total follow-up of four years and five months after the conclusion of the last lengthening procedure (Figure 6).

Currently, there is a persistent Trendelenburg gait to the right and noticeable marked atrophy of the gluteal and quadricep muscles compared to the contralateral side. The lower limbs are symmetrical, and the patient can walk independently. The hips and knees show a full range of motion (ROM), with good patellar stability. The patient is satisfied, even with the complications that occurred during the lengthening.

## 3. Discussion

The surgical treatment of FD poses a challenging task for the surgeon. This is due to the poor bone quality, which increases the difficulty of the surgery and the risk of failure, as well as the high incidence of fractures that these patients experience and the development of complex deformities that negatively impact their quality of life. Surgical indications include pathological fractures, the prevention of recurrences, cases of non-union, and deformities that cannot be adequately corrected with orthotic or conservative treatment [8]. In polyostotic forms, we often encounter weak and thin bones that do not allow us to intervene with a traditional approach. In the literature, there is no absolute consensus regarding the surgical treatment of this condition. Many authors agree that the bone in FD has a significantly lower potential to remodel and to correct residual angulation [12]. Consequently, techniques that minimize ad latus translations of bone segments should be favored, such as lengthening on nail procedures and MILLN [11]. In recent years, the use of intramedullary fixation has been gaining popularity as the first choice of treatment for fracture stabilization in FD [10]; however, few lengthening procedure cases with MILLN were reported in FD [13,18]. The techniques for lengthening severe LLD may involve the use of monaxial, circular, or hexapod fixators or intramedullary nails. The latter allow local complications caused by pins to be avoided, are better accepted by the patient, and reduce the immobilization period [19,20]. The MILLN is one of the most widely used in clinical practice and is extensively described in the literature [14,21,22,23]. It comes in various sizes and can lengthen from 5 to 8 cm depending on the specific nail used [19]. The clinical outcomes are excellent, and patients prefer this treatment over external fixation [20,24]. However, the lengthening intramedullary nails are not without complications. The main ones include implant failure during lengthening, breakage, or the premature consolidation of the osteotomy [20,23]. Due to the limited number of reported cases using MILLN for the treatment of FD, we are unable to determine whether the complications associated with this procedure differ from those in other orthopedic conditions.

In case of treatment failure, several cases have been reported regarding the reutilization of the same nail following a new osteotomy. This procedure is performed in two stages. Initially, only the proximal anchoring screws are removed to allow the nail to self-retract. In the second stage, after radiographically confirming that the intramedullary nail has fully retracted, a new osteotomy can be performed, securing the nail proximally with new screws and resuming gradual lengthening according to the protocol. Cases of MILLN reactivation have been reported, involving both the upper and lower limbs. This procedure has been used both as a planned intervention for significant lengthening and as a salvage procedure following complications [25,26,27,28]. Georgiadis et al. described the reuse of a MILLN in 12 patients. Among these cases, 58% were effectively reactivated. In reactivation cases, however, no major complications were reported [14]. Eltayeby et al. tested 102 consecutively intact explanted nails using a “fast magnet”. In 84.3% of cases, the nails returned within the predefined parameters for lengthening and retraction. The failure to activate the remaining nails was attributed to the complete, and thus excessive, retraction of the nail, which may have damaged the internal mechanism [21]. Alonso-Hernandez et al. reported two cases of failure due to the inability of the MILLN to retract, which were resolved by removing the devices. No cases of major complications have been reported regarding the reactivation of a MILLN [25]. Safi et al. compared the clinical and radiographic results of 21 patients treated with PRECICE and 20 patients treated with FITBONE, demonstrating similar outcomes, excellent cosmesis, and high patient compliance [23]. The literature does not suggest the lengthening nail as the first choice in treating significant leg length discrepancies in individuals with FD. Karakoyun et al. described its use in a single patient [13]. The locking screw (Poller), in combination with the most common lengthening systems, is well described as an almost required surgical step to correct associated deformities and to avoid apparent valgus deformity in femoral lengthening [29,30]. According to our knowledge, there has been no reported case of reusing the same MILLN in a patient with FD. In our clinical case, the patient, following multiple fractures of the lower limbs, was treated to correct an 8.5 cm LLD with significant valgus deformity in the right knee. Our chosen treatment involved using a MILLN, along with a Poller screw. The postoperative course was complicated by the subsidence of the nail with a loss of fixation of the proximal screws. Mechanical failures of the implant, such as screw loosening and nail subsidence, have been reported [20,23]. Given the nature of the adverse event, we cannot definitively state that the patient did not bear weight on the operated limb against medical advice. Furthermore, although the screws appeared to be implanted in a region without obvious dysplasia, we cannot rule out the possibility of underlying dysplastic involvement. The parents and the patient were informed about the possibility of reactivating the already implanted nail. This type of procedure is currently off-label, although it is well reported in the literature. The parents and the patient consented, so the nail was completely retracted and subsequently re-lengthened following a new osteotomy. We measured the Healing Index (HI), according to Teulières et al. [28], as the result of the ratio between the healing period (defined as at least three visible cortical bridges out of four cortices on radiographs and full weight bearing) and the lengthening achieved. After the first lengthening, the HI was 60, after the reactivation of the nail, and the HI was 56. The complications were classified using the Lascombes and Paley classification. According to Paley [31], during the first lengthening, we found an obstacle and not a complication because it was resolved through a surgical reintervention. According to Lascombes [32], we reached grade III-b because our lengthening gained less than the 75% of the initial objective, but no alteration of function was observed. Despite the complications and the limited length achieved by the initial implant compared to the initial goal, we managed to utilize the reactivation of the MILLN to achieve a complete alignment of the lower limbs, avoiding more invasive procedures and reducing management costs. While the reactivation of a MILLN has not been associated with major complications in the cases reported, it is important to note that the use of this technique for off-label indications carries inherent risks. These risks include potential complications from device malfunction or inadequate response to treatment. In our case, these risks were carefully discussed with the patient’s family, emphasizing the need for close monitoring throughout the treatment process and ensuring informed consent. By addressing these concerns upfront and maintaining regular follow-up, the likelihood of complications was minimized, ensuring a positive outcome despite the off-label application. The patient is currently satisfied with the functional outcome, independent of activities of daily living. The main limitations of this study are its retrospective nature and the analysis of a single case. In our opinion, reactivating a MILLN following a mechanical failure offers a less invasive option for a patient who has already experienced multiple fractures and undergone several surgical interventions. Additionally, it provides significant cost savings for the hospital.

## 4. Conclusions

This case report supports the reuse of MILLN in a patient with polyostotic FD. In case of mechanical failure, it enables the equalization of LLD and deformities, avoiding new invasive surgical procedures and reducing healthcare costs. As an off-label treatment, preoperative consent from both the patient and parents is required.

## Data Availability

The data presented in this study are available on request from the corresponding author due to the patient’s privacy.
